# In dogs with aural haematoma does draining and injecting corticosteroids versus drainage alone reduce the risk of recurrence?

**DOI:** 10.18849/ve.v7i1.438

**Published:** 2022-03-31

**Authors:** Rachel Church+

**Keywords:** STRESS

## Abstract

PICO question

In dogs with aural haematomas does draining and injecting corticosteroids versus drainage alone reduce the risk of recurrence?

Clinical bottom line

Category of research question

Treatment

The number and type of study designs reviewed

Three papers were critically reviewed, a retrospective multi-centre cohort study, a randomised case control trial and an observational survey

Strength of evidence

Weak

Outcomes reported

Drainage alone at daily or weekly frequency consistently resulted in aural haematoma (AH) recurrence and lack of resolution. Corticosteroid instillation alongside drainage reduced the risk of rapid recurrence for AHs, across both the cohort and the case control studies, provided drainage was frequent

Conclusion

The strength of evidence for local steroid instillation was weak given the type of studies reviewed, alongside small sample sizes and variations in treatment protocol. However, consistently drainage alone appears an insufficient means of addressing AHs in dogs.

In cases where frequent drainage was the chosen treatment option, the addition of local corticosteroid application appeared to improve the outcome compared to drainage alone.

However, as systemic treatment was often employed alongside local corticosteroid instillation, success cannot necessarily be attributed solely to local treatment. Larger, randomised control trials would be required to assess the effect of each individual intervention providing clearer evidence for the most effective medical protocol for treating aural haematomas in dogs

## How to apply this evidence in practice

The application of evidence into practice should take into account multiple factors, not limited to: individual clinical expertise, patient’s circumstances and owners’ values, country, location or clinic where you work, the individual case in front of you, the availability of therapies and resources.

Knowledge Summaries are a resource to help reinforce or inform decision making. They do not override the responsibility or judgement of the practitioner to do what is best for the animal in their care.

## Clinical scenario

An 11 year old male neutered Labrador Retriever presents to your clinic with an aural haematoma of the right ear. The owner wishes to discuss the options for management without surgical intervention and has asked about the scientific evidence for local treatment of aural haematomas to select the best protocol for the lowest risk of recurrence.

## The evidence

A search of the literature revealed three papers relevant to the PICO. They consist of a retrospective multi-centre cohort study (Mikawa et al., 2005), a randomised non-blinded case control study (Kuwahara et al., 1986b) and an observational survey (Hall et al., 2016).

In the cohort study (Mikawa et al., 2005) aural haematomas (AHs) were managed with frequent drainage alone or drainage and local instillation of corticosteroid (dexamethasone or prednisolone). In all cases which underwent drainage alone the haematoma recurred, requiring additional surgical or medical management to elicit resolution. With repeated drainage and local corticosteroid instillation, all cases resolved within 35 days.

The randomised case control study (Kuwahara et al., 1986b) compared local and systemic corticosteroid treatment of AHs with daily drainage alone. There was an absence of healing in all cases that received daily drainage only. Daily treatment with local and systemic corticosteroids showed satisfactory healing in the majority of cases, and resolution within 5 days.

The observational survey (Hall et al., 2016) detailed the commonly employed treatments for AHs. Drainage with local corticosteroid instillation was more commonly employed for AHs on first presentation than drainage alone, and with improved perceived outcome.

The strength of evidence was weak for the PICO question given the small sample sizes, in addition to variables in the treatment protocols, size and chronicity of the AHs, and the presence of underlying ear and skin disease. Large scale randomised blinded case control studies would be required to provide stronger evidence for the benefit of local corticosteroid instillation over drainage alone.

## Summary of the evidence

**Mikawa et al. (2005):** 

Population:	59 aural haematomas (AHs) in 49 dogs (25 males, 24 females) across five facilities in Miyazaki Prefecture in Japan between January 1998 and March 2005. Mixed breed, with higher incidence of medium to large breed dogs. 22/49 dogs (44.9%) were Golden Retrievers. 45/59 (76.2%) affected ears had concurrent otitis externa. Cases of aural haematoma consolidation or ear deformity were excluded.
Sample size:	23 dogs received interventions specific to the PICO, from a total of 59 AHs in 49 dogs in the full study. The remaining 26 dogs will not be commented on further in this Knowledge Summary.
Intervention details:	AHs were treated conservatively in 23 cases, and surgically in 43 cases (Nine of which were initially treated conservatively). Two cases were untreated. Of those treated conservatively, 14 cases were treated with needle aspiration alone, and nine cases received 0.4% dexamethasone (4–8 mg) or 1% prednisolone (0.5 mg) infusion after needle aspiration. In both groups the treatment was repeated if the haematoma recurred. The treatment endpoint for the group receiving local corticosteroid treatment was AH resolution. The treatment frequency and endpoint in the group undergoing needle aspiration alone were not detailed. The frequency of needle aspiration alongside local corticosteroid instillation was weekly in 5/9 (55.5%) cases. Concurrent treatment for otitis externa in affected cases (based on cerumen examination) was performed alongside AH treatment, the details of which were not supplied.
Study design:	A retrospective multi-centre cohort study.
Outcome studied:	The treatment and progression of each AH was recorded, including whether the AH resolved, if surgical management was subsequently required, if aural deformity occurred, or if the progress was unknown. The duration of treatment was recorded in the group undergoing corticosteroid instillation alongside drainage. In 5/9 cases that received local corticosteroid instillation alongside drainage, recurrence and volume of fluid retention in AH was recorded over a 4 week period. The recurrence of AH was studied with a follow-up period of 2 years in 26 cases, 1–2 years in 13 cases, 6 months to 1 year in 17 cases, and no follow-up in three cases.
Main Findings (relevant to PICO question)	In the 14 cases that underwent drainage alone, none achieved resolution by initial or repeat drainage, with nine then undergoing surgical management. In two cases a total of eight drainage attempts were made. The remaining five cases that were conservatively managed included three that led to aural deformity and two cases that were lost to follow-up. The treatment duration was not stated in the drainage only group. Initial reaccumulation was seen in all nine cases which underwent corticosteroid instillation alongside drainage, but subsequently the AHs resolved in all cases within 35 days. The mean duration of treatment was 18.3 days in the corticosteroid injection group (range 4–35 days). In the five cases receiving corticosteroid instillation in which fluid recurrence was measured, four cases showed a decrease in the level of fluid by week 2 and resolution by week 4, and one case showed an increase until week 3 then a resolution by week 4. This suggests the corticosteroid suppresses the production of haematoma fluid. With corticosteroid instillation, 2/9 (22.2%) cases recurred more than 2 weeks after treatment, 1 and 2 months after treatment had been completed.
Limitations:	The quality of evidence may be reduced by the retrospective and multi-centre nature of the study, with accuracy reliant on the rigor of record keeping. The sample size was small therefore detailed statistical analysis is not possible. There was variability in the chronicity and size of the AHs, and the extent of accompanying ear or skin disease, however, these factors were not detailed across the intervention groups. There was a variable follow-up period. The type and dose of steroids used varied. It was not clear which animals received which dose or type of steroid, and whether this was altered during the management of each case. The drainage frequency in the corticosteroid instillation was weekly in five cases, but unknown in the remainder and in the drainage only group.

**Kuwahara et al. (1986b):** 

Population:	40 dogs and 20 cats seen in a veterinary hospital in Japan, date unknown.
Sample size:	The participants were divided randomly into five groups, of which three relate to PICO, consisting of 21 dogs. The remaining 19 dogs allocated to other groups and the 20 cats will not be commented on further in this Knowledge Summary.
Intervention details:	Group 3 (eight dogs) underwent AH drainage once (complete aspiration performed aseptically with a 16-gauge needle and syringe to remove the dead space of the cavity created by the haematoma). The dogs then received intravenous (IV) dexamethasone 0.5 mg/kg and intramuscular (IM) gentamicin 4 mg/kg. Group 4 (nine dogs) underwent AH treatment consisting of a single flush with sterile saline to remove fibrin, clots and debris. This was followed by daily local instillation of 0.2–0.4 mg dexamethasone (0.2%) and 0.25 mg gentamicin diluted 5–10 times in sterile saline (0.4–1.8 ml) depending upon the extent of the cavity, decreasing to approximately 0.5 ml by the second or third day. Systemic treatment was also given as per Group 3. Group 5 (four dogs) underwent daily AH drainage for 6 days, being flushed with sterile saline and repeat aspiration until the fluid was clear. The saline volume used was 10–50 ml depending on the size of the haematoma. There was no recorded systemic treatment for this group. The treatment end point was defined as complete healing of the lesion. The criteria for a tentative diagnosis of complete healing was an absence of fluid accumulation and clinical signs (e.g. oedema, inflammation, head shaking or ear scratching). Definitive diagnosis was made using the same criteria at re-examination 2 months after the final treatment. All animals were investigated and treated for underlying ear canal disease with a combination of systemic and topical treatments, the details of which are not provided.
Study design:	A randomised non-blinded case control study.
Outcome studied:	Chronicity of AH prior to treatment – acute (0–7 day duration), subacute (8–14 days) or chronic (>15 days). Days required for successful treatment. Satisfaction with healing based on pinnal morphology. A subjective assessment, graded A–D, was made when the AH had healed, in which Grade A denoted no morphological changes to the pinna and Grade D denoted severe changes. Grades A and B were considered to represent satisfactory healing, whereas grades C and D represented unsatisfactory healing. Extent and duration of ear canal disease was assessed based on clinical signs and otoscopic examination. Recurrence of ear canal disease and AH – The results of treatment for otitis externa and AH were recorded on the day following the final treatment, and at follow-up at 2 months and 1 year after the final treatment to assess recurrence.
Main Findings (relevant to PICO question)	Chronicity of AH – The majority of dogs had acute or subacute haematoma development in this study. 7/8 dogs (87.3%) in Group 3 had acute AHs, 1/8 (12.5%) subacute. In Group 4, 8/9 (88.9%) were acute, 1/9 (11.1%) subacute. In Group 5 all dogs had acute AHs. Days required for successful treatment – Dogs in Group 3 had resolution in 100% cases within 9 days. Group 4 dogs showed resolution in 100% of cases within 5 days. Group 5 dogs all showed an absence of healing and subsequently underwent surgical management. Satisfaction with healing based on pinnal morphology. Satisfactory healing (grade A or B) was achieved in 3/8 (37.5%) cases in Group 3, 8/9 (88.9%) cases in Group 4 and no cases in Group 5. In summary Group 4 dogs (single drainage and daily local and systemic treatment) showed satisfactory healing in the majority of cases and with the shortest treatment duration of 5 days. Group 3 dogs (single drainage and systemic treatment) healed within 9 days but fewer achieved satisfactory healing. Extent and duration of ear canal disease – 32/40 (80%) of dogs were suffering from ear canal disease, showing a range of cases from severe otitis externa to mild ear disease affecting only the vertical canal. Recurrence of ear canal disease and AH – there was no recurrence reported at follow-up examination 2 months after the final treatment. After 1 year, recurrence of otitis externa was seen in one case in Group 3.
Limitations:	The paper did not address the impact of local corticosteroid use alone, since systemic corticosteroids were also given. They may affect any associated otitis, but could also exert an effect via suppressing any immune-mediated process that may underlie the development of AHs. Therefore, while it can be inferred that the treatment protocol for Group 4 dogs gave a more favourable outcome to those in Group 5, this success may not be solely attributable to local treatment. The inclusion of Group 3 dogs (those that underwent drainage and systemic corticosteroid treatment) was not directly related to the PICO. However, it has relevance as a comparison to Group 4 which received the same treatment in addition to local corticosteroid instillation, and therefore was included in the analysis. The improved outcome in Group 4 compared to Group 3 may be attributable to the additional local treatment used in this group. In Group 4, gentamicin was injected locally in addition to corticosteroids, and therefore successful outcome may not be entirely attributable to corticosteroid instillation. The severity of disease was detailed clearly but not correlated with individual case outcomes. The volume and concentration of corticosteroid instilled varied depending on the size of the haematoma but this was not correlated with outcome. The study size was small, reducing the power of the study and precluding statistical analysis. The study was randomised but the method was not detailed, and the study was not blinded, with subjective assessment of haematoma healing and resolution of ear disease.

**Hall et al. (2016):** 

Population:	Veterinary surgeons and practices on the RCVS register in the UK and members of the Association of Veterinary Soft Tissue Surgeon (AVSTS), treating small animal patients.
Sample size:	2386 veterinarians were emailed questionnaires, of which 312 email addresses were invalid, and 259 completed questionnaires were received, 259/2074 (12.5%) response rate. Responses were excluded if the questions were not all completed. 251 questionnaires were included in the analysis.
Intervention details:	Veterinarians were asked to select which treatment option they would use for aural haematoma (AH) management at first presentation and for recurrent cases. Treatment options were needle drainage alone, needle drainage with local deposition of corticosteroids, surgical management, placement of a Penrose drain, and ‘other’ procedures. ‘Other’ procedures included systemic corticosteroid treatment with or without needle drainage, needle drainage with local corticosteroid instillation 3–5 days later, medical treatment of otitis externa for 7–10 days before needle drainage with local administration of corticosteroids, bandaging and the haematoma being left to resolve naturally. There were no details provided regarding corticosteroid doses or frequency of drainage.
Study design:	Observational subjective survey.
Outcome studied:	Respondents were questioned to establish the preferred treatment for AHs on initial presentation, for recurrent or persistent haematomas and for multiple recurrent haematomas. The reason for the treatment selection was recorded (previous success, owner preference, cost, practice policy, convenience, other). Perceived success of the treatment (prevention of recurrence, cosmetic outcome and predicted owner satisfaction) were subjectively rated by the respondents as excellent, good, satisfactory or poor. Questions relating to the treatment of skin and ear disease were not included in the questionnaire.
Main Findings (relevant to PICO question)	For the management of AHs at initial presentation, 109/251 (43%) of respondents used drainage with local instillation of corticosteroids, compared to 40/251 (16%) who used drainage only. 20/251 (8%) were in the ‘other’ category, which included elements relevant to the PICO but was not detailed to allow further evaluation. Recurrent haematomas were treated by needle drainage with local instillation of corticosteroids in 40/251 (16%) of cases, compared to needle drainage alone in 17/251 (7%) of cases. 168/251 (67%) cases underwent surgical management. In cases with multiple recurrence 12/251 (5%) underwent drainage and local steroid instillation, and 5/251 (2%) received needle drainage alone. 161/251 (64.3%) underwent surgical management. Perceived success – 13/109 (12%) respondents stated excellent perceived success with corticosteroid instillation and drainage, 51/109 (47%) good, 33/109 (30%) satisfactory and 12/109 (11%) poor. In comparison, 5/40 (13%) stated excellent perceived outcome with needle drainage alone, 10/40 (25%) good, 22/40 (56%) satisfactory and 3/40 (8%) poor. Expected recurrence – 56/109 (51%) of respondents who used local instillation of corticosteroids alongside drainage as a first line treatment expected recurrence. There was no comparison figure for drainage only. Cosmetic outcome – this was stated as excellent for medical management and good for surgical management. Owner satisfaction with chosen treatment option – stated as excellent 41/251 (16%) of cases, 141/251 (56%) good, 65/251 (26%) average, 4/251 (1.5%) poor. Respondents’ reasons for the treatment choice – 193/251 (77%) made their choice based on previous success, followed by owner preference 15/251 (6%), cost 13/251 (5%), practice policy 10/251 (4%), convenience 10/251 (4%) and other 10/251 (4%). 153/251 (61%) respondents expected 0–25% chance of recurrence with their first treatment choice.
Limitations:	This was a weak form of evidence as it was an observational and subjective study, based on previous experience and perceived success of participants, therefore subject to substantial bias. There was objective assessment by the authors collating the results, but based on subjective assessment of AH by participants. The study lacked description of the size and extent of the AHs, which could affect treatment options chosen and therefore their predicted success. Concurrent treatment for skin or ear disease was felt to be mandatory by the authors, but not detailed in the questionnaire, which made the findings difficult to review, as systemic treatments such as corticosteroids may have been used concurrently. The predictions of recurrence across different treatment options were not clearly displayed, neither was the time frame for recurrence. There were limitations relating to a survey with potential for misinterpretation of the questions, bias towards those with a technological disposition due to its distribution only through email, and bias towards a subset of practitioners more inclined to complete such surveys. The inclusion of AVSTS members may also bias towards surgical options or more chronic cases. The ‘other’ category included use of local instillation of steroids delayed from the initial drainage, but no further details were provided to allow evaluation of this subset.

## Appraisal, application and reflection

Aural haematomas (AHs) are caused by pinnal cartilage rupture resulting in serosanguinous fluid entering the cavity. However, their pathogenesis in dogs remains unclear and is likely to be multifactorial, with theories relating to trauma, an immune mediated process (Kuwahara et al., 1986a; and Lahiani & Niebauer, 2020), and cartilage degeneration with involvement of immunological factors (Joyce & Day, 1997).

There are numerous treatment options for AHs in dogs, with a wide range of medical and surgical techniques described (Ahirwar et al., 2007; and Swaim & Bradley, 1996). The above putative causes have prompted the use of local corticosteroid instillation as a treatment option, while ensuring a sterile technique is employed, as corticosteroids can predispose to abscess formation (Seibert & Tobias, 2013). However, as concurrent otitis externa and allergic skin disease are common (MacPhail, 2016), systemic corticosteroids are frequently used alongside local AH management, complicating the analysis of local corticosteroid treatment efficacy (Joyce, 1994).

Three papers have been found which provide weak evidence towards the use of local instillation of corticosteroids in canine AHs in comparison to drainage alone. Drainage techniques beyond needle aspiration and flushing have not been included. These would be better assessed with a PICO focusing on the comparison of different drainage techniques. Furthermore, needle aspiration and local corticosteroid instillation can be performed in the conscious patient, with direct relevance to situations in clinical practice where chemical restraint would be best avoided, such as older patients or those with concurrent disease.

The randomised control trial (Kuwahara et al., 1986b) is the preferred study design for assessing treatment. The trial showed recurrence in all cases of AH treated with drainage alone, necessitating either surgical intervention or alternative conservative management. Therefore, in cases where surgical management is not appropriate or suitable, the results of this trial suggest that a treatment protocol including daily steroid instillation alongside drainage would be more successful than drainage alone in preventing recurrence and achieving resolution.

The study did not, however, provide a clear comparison of local instillation of corticosteroid to daily drainage alone, since systemic treatment was used in addition to local corticosteroid treatment. Therefore, the outcome could not be solely attributed to the local treatment. Furthermore, gentamicin was also instilled locally alongside corticosteroids and so the success of local treatment may not have been solely attributable to corticosteroid instillation in these cases.

The study was randomised which improves the strength of evidence when assessing treatment interventions.  However, the outcomes were not matched to the severity of the disease, the sample size was small, and the amount of steroid instilled was variable. Larger studies would be required to analyse this further and guide treatment selection based on the individual patient.

The retrospective cohort study (Mikawa et al., 2005) provides a moderate level of evidence for assessing treatment. It revealed that repeated aspiration and instillation of corticosteroids was more effective than aspiration alone, with all AHs being resolved over a period of 35 days. In comparison, drainage alone did not produce resolution of the AH in any of the 14 cases, in spite of additional systemic treatment in all cases with underlying ear and skin disease.

However, the sample size was small and the population was not randomised. There was also variation in the amount and type of corticosteroid instilled, and the severity or size of the AH was not detailed, all of which may influence the results. Frequent repeated treatment was required in many cases, therefore consideration should be given as to which patients would be most appropriate to manage in this manner. The frequency of treatment was variable and not clearly defined in all cases. This paper highlights a need for further studies to evaluate alternative corticosteroid preparations and frequencies, including longer acting preparations that could be instilled less frequently.

The observational survey (Hall et al., 2016) is a weak form of evidence for addressing the research question. It revealed a majority of practitioners used corticosteroid instillation alongside AH drainage at first presentation, but 56/109 (51%) predicted recurrence. This study concurred with the findings of the previous two papers that drainage alone was not sufficient for resolution, but also suggested that local corticosteroid treatment with or without systemic corticosteroids would not be adequate in some cases.

The observational survey was, however, subjective and affected by respondent bias and variables relating to their experience, severity of the AH and / or the presence of underlying ear disease. Furthermore, additional treatments for concurrent disease were not detailed but would be expected to influence the treatment outcomes. The frequency of drainage or corticosteroid instillation were also not discussed, though such variations in the frequency and type of treatment employed in conservative management of AHs in clinical practice would be likely to influence the outcome.

Across the three papers studied, there was evidence to suggest that drainage alone had a high recurrence rate, prompting the need for alternative treatment options. The use of local instillation of corticosteroids as part of alternative protocols was associated with an improved outcome. However, the small sample sizes and variables across the papers meant that assessment of whether it was the sole reason for the improved outcome was less clear. Large scale randomised control trial studies would therefore be required to evaluate this further.

## Methodology Section

**Search Strategy:** 

Databases searched and dates covered:	CAB Abstracts on the OVID interface 1973–2021 Week 47 PubMed accessed via the NCBI website 1920–December 2021
Search strategy:	CAB Abstracts: (dog or dogs or canine or canines or canis).mp. or exp dogs/ or exp canis/ (aural or ear* or pinna* or auric*).mp. and ((haematoma* or hematoma* or othaematoma or othematoma).mp. or exp haematoma/) (corticosteroid* or corticoid* or glucocorticoid* or steroid* or dexamethason* or dexadreson or dexafort or 'triamcinolone acetonide' or methylprednisolone acetate or depomedrone or prednisolone).mp. or exp glucocorticoids/ or exp steroids/ (Drain* or remov* or fluid or aspirat* or nonsurg* or non-surg* or 'non surg*' or suction*).mp. 1 and 2 and (3 or 4) PubMed: Dog or canine or canis (aural or ear or ears or pinna or auricular or auris) and (haematoma or hematoma or othaematoma or othematoma) corticosteroid or corticoid or glucocorticoid or steroid or dexamethasone or dexamethason or dexadreson or dexafort or 'triamcinolone acetonide' or methylprednisolone acetate or depomedrone or prednisolone drain or drainage or remove or fluid or aspirate or nonsurgical or non-surgical or “non-surgical” or suction 1 and 2 and (3 or 4)
Dates searches performed:	02 Dec 2021

**Exclusion / Inclusion Criteria:** 

Exclusion:	Irrelevant to PICO Not in English language Only one technique included Surgical management Not canine patients Review articles, non-peer reviewed material, conference proceedings Duplicates
Inclusion:	Peer-reviewed material English language Comparative papers including both aural haematoma drainage with corticosteroid injection and aural haematoma drainage alone Canine patients

**Search Outcome:** 

Database	Number of results	Excluded – Irrelevant to PICO	Excluded – Non-English	Excluded – Not comparing specified techniques	Excluded – Surgical management	Excluded – Not canine	Excluded – Not peer-reviewed, conference or review paper	Total relevant papers
CAB Abstracts	55	15	3	11	14	4	5	3
PubMed	110	97	0	7	1	0	4	1
Total relevant papers when duplicates removed								3

## Conflict of Interest

The author declares no conflicts of interest.

The search was developed by Clare Boulton.

## Disclaimer

Knowledge Summaries are a peer-reviewed article type which aims to answer a clinical question based on the best available current evidence. It does not override the responsibility of the practitioner. Informed decisions should be made by considering such factors as individual clinical expertise and judgement along with patient’s circumstances and owners’ values. Knowledge Summaries are a resource to help inform and any opinions expressed within the Knowledge Summaries are the author’s own and do not necessarily reflect the view of the RCVS Knowledge. Authors are responsible for the accuracy of the content. While the Editor and Publisher believe that all content herein are in accord with current recommendations and practice at the time of publication, they accept no legal responsibility for any errors or omissions, and make no warranty, express or implied, with respect to material contained within. For further information please refer to our Terms of Use.
